# Gender Trends in Professional Advancement Among Academic Pediatric Neurologists

**DOI:** 10.1001/jamanetworkopen.2025.40884

**Published:** 2025-10-31

**Authors:** Juliet K. Knowles, Angela L. Hewitt, Prathyusha Teeyagura, Bren Botzheim, Derek Boothroyd, Ria Pal, Chrisoula Cheronis, Shermila Pia, Rebecca MacRae, Christine Shrock, Taelor Hancock, Rayann Solidum, Jaclyn Peraino, Amy Hill, Laura Owlett, Sara N. Moss, Catherine Tran, Daniel A. Castillo, Mark S. Wainwright, Renée A. Shellhaas, Nomazulu Dlamini, Mustafa Sahin, Rose Gelineau-Morel, Joshua L. Bonkowsky

**Affiliations:** 1 Department of Neurology, Stanford University School of Medicine, Stanford, California; 2 Department of Neurology, University of Rochester School of Medicine, Rochester, New York; 3 Quantitative Sciences Unit, Stanford University School of Medicine, Stanford, California; 4 Department of Neurology, Children’s Hospital of Los Angeles, Los Angeles, California; 5 Department of Neurology, Boston Children’s Hospital, Harvard Medical School, Boston, Massachusetts; 6 Department of Pediatrics, University of Utah School of Medicine, Salt Lake City; 7 Department of Pediatrics, Children’s Mercy Hospital, University of Missouri-Kansas City School of Medicine, Kansas City; 8 University of Rochester Libraries, University of Rochester, Rochester, New York; 9 Division of Pediatric Neurology, University of Washington School of Medicine, Seattle; 10 Department of Neurology, Washington University School of Medicine, St Louis, Missouri; 11 Division of Neurology, Department of Paediatrics, Hospital for Sick Children, University of Toronto, Toronto, Ontario, Canada; 12 Center for Personalized Medicine, Primary Children’s Hospital, Salt Lake City, Utah

## Abstract

This cross-sectional study examines gender representation and metrics of academic success for academic pediatric neurologists who were board certified between 2000 and 2020.

## Introduction

In the US, women comprise more than 50% of medical school graduates^1^ and roughly half of the residency workforce,^2^ but are underrepresented among medical school faculty.^3^ Equal gender representation is associated with improved patient satisfaction, quality of care, and research innovation.^4,5^ Gender trends have not been systematically studied in academic pediatric neurology. We quantified gender representation and metrics of academic success for academic pediatric neurologists who were board certified between 2000 and 2020.

## Methods

Complete methods for this cross-sectional study are available in the eAppendix in Supplement 1. The Stanford University institutional review board review deemed this study exempt from review, given that all data collected for the study were accessed from publicly available databases. This study follows the STROBE reporting guideline. We defined academic pediatric neurologists as holding an active faculty position at a US medical school. Data were collected between February and September 2023 for all pediatric neurologists who were first board certified from 2000 to 2020. Predetermined outcomes were related to publications, National Institutes of Health grants, and academic rank. Cumulative publications through 2023 were included regardless of year of board certification. Data were analyzed in aggregate (diplomates certified 2000-2020) or using 5- to 6-year cohorts (those board certified between 2000-2005, 2006-2010, 2011-2015, and 2016-2020) as a stratifying variable, because metrics are expected to vary with time since board certification. The dataset comprised 21 years (2000-2020); thus, 1 cohort included 6 years (2000-2005). Interrater reliability analysis was performed on 200 individuals board certified from 2000 to 2020 (eTable in Supplement 1); outcomes with strong interrater agreement are reported. We calculated the interclass correlation for continuous outcomes, the κ statistic for categorical outcomes, and weighted κ for ordinal outcomes. *P* < .05 was considered statistically significant. R statistical software version 4.2.1 (R Project for Statistical Computing) was used for the analyses.

## Results

Of 1953 board-certified pediatric neurologists, 1891 met inclusion criteria. From 2000 to 2020, 1126 of 1891 board-certified pediatric neurologists were women (59.5%) with women making up the majority of academic pediatric neurologists (713 of 1136 neurologists [62.7%] beginning in 2006 to 2010) (Table). The proportion of academic pediatric neurologists who were women increased over time (69 of 139 academic pediatric neurologists [48.9%] certified from 2000 to 2005; 278 of 413 [67.3%] certified from 2016 to 2020) (Table).

**Table.** Outcomes by Cohort and Gender

Variable	Cohort 1 (2000-2005)		Cohort 2 (2006-2010)		Cohort 3 (2011-2015)		Cohort 4 (2016-2020)		Overall (2000-2020)	
	Participants, total No. (% women)	*P* value	Participants, total No. (% women)	*P* value	Participants, total No. (% women)	*P* value	Participants, total No. (% women)	*P* value	Participants, total No. (% women)	*P* value
All pediatric neurologists	273 (45.4)	.41	455 (54.1)	.85	519 (64.7)	.63	644 (65.2)	.95	1891 (59.5)	<.001
Academic pediatric neurologists only	139 (48.9)		264 (56.8)		320 (67.8)		413 (67.3)		1136 (62.7)	
No. of publications, median (IQR)										
Men	79.1 (15.0-143.0)	.13	53.7 (9.0-76.0)	.005	31.1 (7.0-40.0)	.001	18.2 (5.0-19.8)	.001	41.3 (7.0-49.3)	<.001
Women	59.8 (15.5-88.5)		35.4 (7.0-44.5)		21.0 (5.0-25.2)		12.7 (4.0-16.0)		24.5 (5.0-27.5)	
h-Index, median (IQR)										
Men	19.3 (6.0-29.0)	.13	14.1 (4.0-22.0)	.001	8.8 (3.0-12.2)	.001	5.5 (2.0-7.0)	.02	11.0 (3.0-15.0)	<.001
Women	15.5 (5.5-23.5)		9.3 (3.0-13.0)		6.3 (2.0-9.0)		4.3 (1.0-6.0)		7.0 (2.0-10.0)	
First or last author publications, median (IQR), %										
Men	21.9 (16.0-30.0)	.26	22.3 (16.0-27.5)	.14	21.5 (13.5-27.6)	.07	20.3 (12.5-28.2)	.99	21.4 (14.5-28)	.04
Women	19.8 (12.5-26.2)		20.1 (10.9-28)		18.9 (12.0-25.6)		20.3 (11.0-28.0)		19.8 (11.5-27.5)	
Role^a^										
Instructor or other	2 (<0.1)	.12	27 (55.6)	.03	22 (81.8)	.07	37 (75.7)	.15	88 (69.3)	<.001
Assistant professor	25 (52.0)		85 (65.9)		203 (69.0)		352 (66.8)		665 (66.8)	
Associate professor	51 (62.7)		100 (58.0)		90 (63.3)		21 (57.1)		262 (60.7)	
Professor	58 (37.9)		52 (40.4)		5 (40.0)		0		115 (39.1)	

^a^ Data were not available for 6 individuals.

Regarding the cumulative publication record as of 2023 for academic pediatric neurologists board certified from 2000 to 2020, men had more publications than women (incidence rate ratio, 1.45; 95% CI, 1.27-1.65; *P* < .001) (Figure, panels A and B). The mean (SD) number of publications for men compared with women was 41.3 (58.9) vs 24.5 (36.9) (Figure, panels A and B; Table). The h-index was higher for men than women, and a higher percentage of men had first or last author position (Table). Men had a higher rank than women (odds ratio, 1.57; 95% CI, 1.22-2.02; *P* < .001); the odds of men ranking higher than women were numerically similar between cohorts (Figure, panels C and D; Table).

**Figure.**
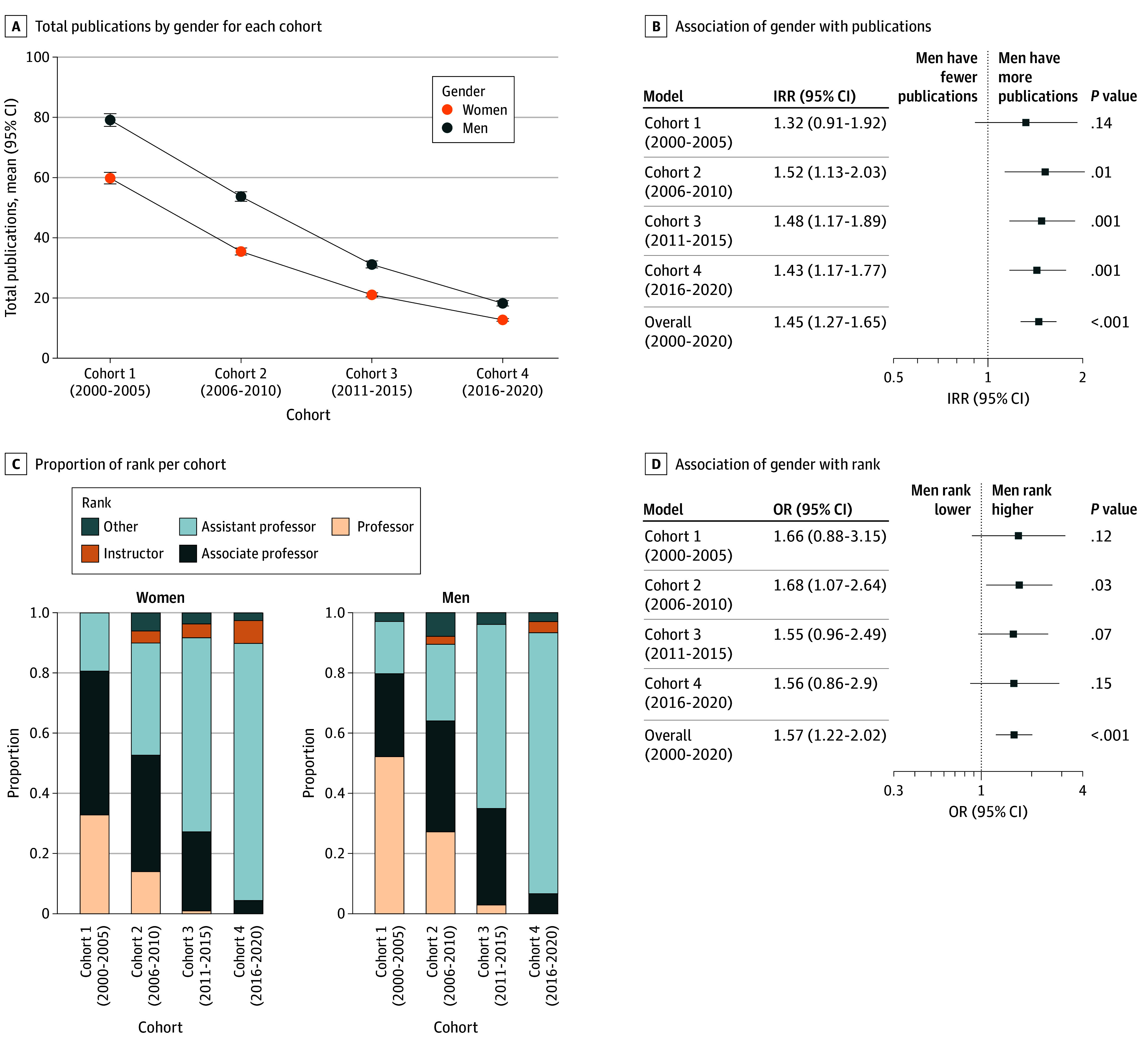
Publication and Rank Findings by Gender A, Mean total number of publications by gender for each cohort between 2000 and 2020. Error bars denote 95% CIs. B, Relative risk (incidence rate ratio [IRR]) that men have more publications than women by cohort. C, Proportion of women or men in each rank, in each cohort. D, Odds (odds ratio [OR]) that men are more highly ranked than women, by cohort.

## Discussion

In this cross-sectional study, we found that women lag men across multiple metrics of academic success in academic pediatric neurology, similar to other medical specialties. This was somewhat unexpected in pediatric neurology, where women constitute the majority of new academic pediatric neurologists since 2006. Our findings indicate that gender differences may not necessarily resolve as a natural consequence of the increased proportion of women entering a specialty, in this case, pediatric neurology.

Strengths of this study include a systematic approach to data collection that avoids selection bias. Limitations include that data were limited to the US, potentially limiting generalizability; additional metrics of academic success (eg, leadership positions and teaching achievements) and potentially influential factors (eg, race and ethnicity and number of dependents) were not publicly available.

Factors including bias, harassment, lack of women role models, lack of representation in major awards and leadership positions, lack of sponsorship,^6^ increased domestic workload, and others may contribute to gender differences in academic medicine. These differences may be damaging for both women and men, although the extent might differ.^6^ Systematic approaches to identify root causes and implement corrective strategies with measurable outcomes have been suggested for academic medical centers, funding agencies, journals, and medical societies^6^; such strategies could be beneficial in academic pediatric neurology.

## Notes

Supplement 1. eAppendix. Supplemental methods eTable. Inter-rater reliability (IRR) analysis eReference

Supplement 2. Data Sharing Statement
