# Supplementary material for: Gender Trends in Professional Advancement Among Academic Pediatric Neurologists
Source: JAMA Netw Open. 2025 Oct 31;8(10):e2540884. doi: 10.1001/jamanetworkopen.2025.40884 (PMC12579340; doi:10.1001/jamanetworkopen.2025.40884)
Supplement: Supplement 1. — eAppendix. Supplemental methods eTable. Inter-rater reliability (IRR) analysis eReference [file jamanetwopen-e2540884-s001.pdf]

Supplemental Online Content

Knowles JK, Hewitt AL, Teeyagura P, et al. Gender trends in professional advancement among academic pediatric neurologists, 2000-2020. *JAMA Netw Open*. 2025;8(10):e2540884. doi:10.1001/jamanetworkopen.2025.40884

eAppendix. Supplemental methods

eTable. Inter-rater reliability (IRR) analysis

eReference

This supplemental material has been provided by the authors to give readers additional information about their work.

© 2025 Knowles JK et al. *JAMA Network Open*.

eAppendix. Supplemental Methods

Institutional Review Board (IRB) review determined that the study was exempt from IRB approval, given that all data collected for the study were accessed from publicly available databases. We followed STROBE guidelines for a cross-sectional study (https://www.goodreports.org/reporting-checklists/strobe-cross-sectional/). To avoid sampling bias, we conducted a comprehensive study of all ABPN diplomates who were board certified in Neurology with Special Qualifications in Pediatric Neurology between the years 2000-2020. A comprehensive list of pediatric neurologists certified between those dates, along with the year of board certification, was obtained with permission from the ABPN. To mitigate observer bias among co-authors contributing to data collection, we relied on objective, publicly available measures in each domain as described below.

We defined academic pediatric neurologists as holding an active faculty position (e.g. Instructor, Assistant Professor, Associate Professor, Full Professor) at a United States medical school, with documentation of their faculty position on an academic medical center website at the time of data collection. Only pediatric neurologists practicing in the United States were included, given that metrics of academic success can differ between countries. Individuals who were deceased or without any public record of professional activity relevant to the field of pediatric neurology were also excluded. Of 1,953 board certified pediatric neurologists, the number of pediatric neurologists meeting criteria for study inclusion was 1,891, of whom 1,136 (60%) met the definition of academic pediatric neurologists. 62 were excluded from analysis because they were deceased (N=13), practicing outside the US (N=38) or retired/not practicing pediatric neurology (N=11).

Data were collected between February to September 2023. Gender (man or woman) was obtained via individual National Provider Identifier (NPI) profiles

© 2025 Knowles JK et al. *JAMA Network Open*.

(https://npiregistry.cms.hhs.gov/search). During the period of data collection and until April 2024, gender on NPI profiles was listed exclusively as male or female, without other gender identity codes.[1] Academic rank (Instructor, Assistant, Associate or Full Professor) was obtained from medical school websites; degrees earned (including medical degrees, MBBS, DO, MD, and additional degrees such as master's or PhD degrees) were recorded from diplomate records, NPI searches and medical school website profiles. Metrics related to publication record were obtained from the Web of Science (WOS), including total publications, h-index, and percentage of publications as first or last author. WOS profiles that were found to contain errors were referred to an academic librarian (D.C.), J.K.K. and A.L.H. for joint review. When review determined that > 2 publications were incorrectly indexed in WOS for a given author, manual quantification of publication metrics was performed in WOS by J.K.K and A.L.H. NIH grant funding data were obtained from NIH reporter (https://reporter.nih.gov/), including grant type (K-series, R01, other), the first year of K-series or R01 funding, and whether NIH funding was active.

Statistical analysis

Inter-rater reliability analysis

Data were entered into a REDCap database for analysis. For a randomly selected group of 200 individuals (~10% of the database), a second group of authors independently collected a duplicate set of data for an inter-rater reliability analysis. The individuals whose data were used for the inter-rater reliability analysis had board certification dates spread evenly across 2000-2020. Inter-rater reliability analysis was performed on endpoints relevant to all individuals (academic and non-academic) in the dataset, including gender, current academic status, and medical degree (MD, DO, MBBS, other), **eTable 1**. Where relevant, inter-rater reliability analysis

© 2025 Knowles JK et al. *JAMA Network Open*.

was performed for endpoints using only data for the subset of diplomates who were counted as academic (related to publications, NIH grant funding and rank). We calculated the interclass correlation (ICC) for continuous outcomes, the kappa statistic for categorical outcomes, and weighted kappa for ordinal outcomes. Outcomes with the strong interrater agreement (kappa or intra-class coefficient > 0.7) are reported. In preliminary studies, we found that accurate classification of an individual as "academic" or "nonacademic" required knowledge of which centers qualify as academic or not. Therefore, this information was collected by pediatric neurologists from the author list. Throughout, a p-value <0.05 was considered to be indicative of statistical significance.

Analysis of primary and secondary endpoints

Primary and secondary endpoints in each of the domains assessed (related to publication records and academic rank) were determined prior to data collection. Given that we found no differences in gender proportions within each cohort (**Table 1**), we included cohort as a covariate in statistical models described below. Missing data were rare, < 30 individuals per variable, and were excluded from analyses.

Publication record: The primary outcome for publication record was the total number of publications. For our primary analysis, a negative binomial model was fitted to investigate the effect of gender on total publications. The secondary outcomes were h-index score and combined percentage of publications in which men or women were the first or last author. We fit linear regression models to investigate the effects of gender on both of these secondary outcomes, including cohort group as a covariate.

Academic rank: The primary outcome for academic rank was the job title (e.g. Instructor, Assistant, Associate or Full Professor). To investigate the potential interaction between gender

© 2025 Knowles JK et al. *JAMA Network Open*.

and academic rank, we fit an ordinal, logistic regression model with cohort group as a covariate. An ordinal analysis incorporates the ranking structure where 'Assistant Professor' is a higher rank than 'Instructor' but a lower rank than 'Professor', thus we could investigate not only whether men and women were likely to receive different academic titles, but also whether men were more likely to achieve a higher academic rank than women.

Cohort analyses: To probe whether gender differences in metrics of academic success vary between successive cohorts, individual models were fit to each cohort (2000-2005, 2006-2010, 2011-2015, 2016-2020). We repeated the above analyses on each individual cohort group, with gender as the only covariate. Since the data were collected as a current snapshot, with length of time certified as one of the only temporal variables, this allowed us to descriptively investigate if there are changing trends over time.

Data availability statement

Data generated from this study will be made freely available upon reasonable request.

© 2025 Knowles JK et al. *JAMA Network Open*.

eTable: Inter-rater reliability (IRR) analysis

	Variable	IRR
**Categorical Analyses** *Kappa*	Gender (Woman/Man)	0.99 (0.97 – 1)
	Current Academic (Y/N)	0.93 (0.88 – 0.99)
	R01 grant (Y/N)	0.65 (0.29 – 1)
	K grant (Y/N)	0.71 (0.41 – 1)
	Active funding (Y/N)	0.59 (0.28 – 0.91)
	Other NIH grant (Y/N)	0.67 (0.42 – 0.92)
**Ordinal Analyses** *Weighted Kappa*	Medical degree (MD, DO, MBBS)	0.95 (0.88 – 1)
	Rank (Instructor, Assistant, Associate, or Full Professor or Other)	0.83 (0.71 – 0.94)
	K-series grant start year	0.67 (0.16 – 1)
**Continuous Analyses** *Intra-class coefficient*	Last author publications, %	0.97 (0.94 – 0.98)
	First author publications, %	0.96 (0.93 – 0.98)
	H-index	0.93 (0.89 – 0.96)
	Total number publications	0.73 (0.59 – 0.83)

eReference

1. Available at: https://download.cms.gov/nppes/NPI_Files.html. Accessed September 29, 2024.

© 2025 Knowles JK et al. *JAMA Network Open*.
