# Supplementary material for: Gender Trends in Professional Advancement Among Academic Pediatric Neurologists
Source: JAMA Netw Open. 2025 Oct 31;8(10):e2540884. doi: 10.1001/jamanetworkopen.2025.40884 (PMC12579340; doi:10.1001/jamanetworkopen.2025.40884)
Supplement: Supplement 2. — Data Sharing Statement [file jamanetwopen-e2540884-s002.pdf]

Data Sharing Statement

Knowles. Gender Trends in Professional Advancement Among Academic Pediatric Neurologists, 2000-2020. *JAMA Netw Open*. Published October 31, 2025. doi:10.1001/jamanetworkopen.2025.40884

Data
Data available: Yes
Data types: Participant data with identifiers
How to access data: jkk1@stanford.edu
When available: With publication

Supporting Documents
Document types: None

Additional Information
Who can access the data: Academic researchers, upon reasonable request.
Types of analyses: For academic research purposes.
Mechanisms of data availability: Data are publicly available already; we merely compiled them into a database.
